# Effects of grazing on vegetation diversity and soil multifunctionality in coconut plantations

**DOI:** 10.3389/fpls.2022.1109877

**Published:** 2023-01-11

**Authors:** Qianwen Duan, An Hu, Weibo Yang, Ruoyun Yu, Guodao Liu, Hengfu Huan, Rongshu Dong, Xinyong Li

**Affiliations:** ^1^ Tropical Crops Genetic Resources Institute, Chinese Academy of Tropical Agricultural Sciences, Haikou, China; ^2^ Key Laboratory of Crop Gene Resources and Germplasm Enhancement in Southern China, Ministry of Agriculture & Rural Affairs, Haikou, China; ^3^ Key Laboratory of Tropical Crops Germplasm Resources Genetic Improvement and Innovation of Hainan Province, Haikou, China; ^4^ Coconut Research Institute of Chinese Academy of Tropical Agricultural Sciences, Wenchang, China

**Keywords:** geese grazing, tropics, taxonomic diversity, functional diversity, soil functions

## Abstract

Grazing is the main way of utilizing understory vegetation in the tropics. However, the effects of grazing on vegetation diversity and soil functions in coconut plantations remain unclear. Therefore, this study was conducted in a young coconut plantation that was grazed by geese in Wenchang, China. We identified four grazing intensities according to the aboveground biomass, namely, no grazing (CK), light grazing (LG), moderate grazing (MG), and heavy grazing (HG). In April 2022, we used the quadrat method to investigate the composition and traits of vegetation, collected and analyzed 0–40-cm soil samples in each grazing intensity. The results showed that grazing changed the composition of understory species. The predominant species changed from *Bidens pilosa* to *Praxelis clematidea* + *Paspalum thunbergii* and then to *P. clematidea* with increasing grazing intensity. The richness, Shannon-Wiener index, evenness, modified functional attribute diversity (MFAD), functional divergence (Fdiv), and functional evenness (Feve) of CK were 4.5, 1.0, 0.29, 0.20, 0.84, and 0.80, respectively. Taxonomic diversity did not respond to LG, but responded significantly to MG and HG. Compared with CK, MG and HG increased richness by 96% and 200%, respectively, and Shannon-Wiener index increased by 40% and 98%, respectively. HG increased evenness by 95%. For functional diversity, MG and HG increased MFAD by 164% and 560%, respectively, but Fdiv and Feve did not respond to grazing intensity. The carbon (C) functioning, nitrogen (N) functioning, phosphorus (P) functioning, and multifunctionality in the 0–10-cm topsoil of CK were −0.03, 0.37, −0.06, 0.20, and 0.14, respectively. Grazing increased C functioning, P functioning, and multifunctionality in the 0–10-cm topsoil but decreased N functioning. Multiple linear regression showed that the taxonomic diversity and functional diversity could be used to estimate soil functions, but these vary among soil layers. In general, MG and HG can increase vegetation diversity and soil function. It may be possible to promote even distribution of geese by adding water sources or zoning grazing. Furthermore, quantitative grazing experiments are needed to determine the efficient use pattern of the understory in coconut plantations in tropics.

## 1 Introduction

Grazing is the most economical and ecological management scheme for utilization of grassland resources (Vallentine, 2001; Hou and Yang, 2006). It is also one of the most important ways of managing terrestrial ecosystems (Kopetz, 2013). Grazing animals mainly act on grassland by feeding, trampling and excreta, and the intensity of grazing animals is higher than that of wild herbivores (Barthelemy et al., 2019). Grazing in agroforestry systems can effectively control weeds (Tohiran et al., 2017) and change community structure, species diversity (Carmona et al., 2012; Pulungan et al., 2019), plant-soil interactions, and soil function (Soliveres and Eldridge, 2014). Many studies conform with the moderate-disturbance hypothesis (Fox, 1979) that moderate grazing increases species diversity, while heavy grazing does the opposite (Herrero-Jáuregui and Oesterheld, 2018; Wang et al., 2018). In California, cessation of grazing decreased native species richness in grasslands, in relation to topography (Gornish et al., 2018). The medium-low stocking rate had no effect on species richness and diversity, while medium-high stocking rate had a negative effect on species richness (Pizzio et al., 2016). However, Dorrough and Scroggie (2008) found that heavy grazing improves grassland species diversity. In Hungary, cattle grazing increased grassland species richness, and livestock species are more important than grazing intensity (Tóth et al., 2018).

Functional diversity can represent the role of plant individual in the ecosystem function (Ford et al., 2018). It is also an important indicator for studying the structure and function of ecosystems. The effect of grazing on plant functional diversity is mainly through “environmental filtering”, which selects the convergent trait values of the species expected to coexist, thus leading to the loss of some functional diversity (Kraft et al., 2008; Ford et al., 2018). Many studies on the effects of herbivore grazing on functional diversity in forest ecosystem have focused on the understory grassland community, and the results are quite different. Some studies found that functional diversity is positively correlated with grazing intensity (Mandle and Ticktin, 2015), while others observed that these were negatively correlated (de Bello et al., 2006). The functional diversity response to livestock grazing is also inconsistent in the grassland ecosystem (Catorci et al., 2014; Komac et al., 2015).

For soils, the aboveground and belowground parts of plants are the main sources of soil organic carbon (SOC), which determine the quality and quantity of litter and roots (McSherry and Ritchie, 2013). Grazing affects soil physicochemical properties through direct disturbances, such as trampling and nutrient addition from feces inputs (Barthelemy et al, 2019). Soliveres and Eldridge (2014) suggested that as the grazing intensity increased, soil functioning decreased. Peco et al. (2017) found that the effect of grazing on soil functioning depends on primary productivity. Soil functioning is closely related to the composition and diversity of aboveground vegetation. However, the effects of grazing on plant, soil, and their interactions are complex (Herrero-Jáuregui and Oesterheld, 2018). The effects of grazing on grassland biodiversity are closely related to grazing history, grazing system, livestock species, grassland type, precipitation, and scale (Reitalu et al., 2010; Vermeire et al., 2018; Gao and Carmel, 2020; Rahmanian et al., 2020).

Coconut (*Cocos nucifera* L.) plantations are common in the wet tropics, ranging from 23° south to 23° north, mostly in coastal areas of Africa, Latin America, and Asia (Moore and Howard, 1996). Hainan Province is the main coconut producing area in China, accounting for 99% of total coconut production (Lu et al., 2021). Coconut plantations are characterized by taller coconut trees, greater spacing between trees, and fewer leaves with smaller shade, resulting in a larger available area in the plantations. To make full use of the open space under the coconut forest, cash crops, crops, or green manure crops are often intercropped (Osei-Bonsu et al., 2002; Ginigaddara et al., 2016). Although some economic income could be obtained, these crops require more investment and labor costs. In addition, it can also lead to various ecological problems, such as reduced diversity of understory vegetation, reduced soil fertility, reduced aboveground vegetation cover, and increased risk of soil erosion, leading to an unstable and unsustainable coconut-forest intercropping system (Adamala et al., 2019). Water and heat conditions are better in the tropics, and the growth rate and growth period of herbage are longer. Grazing is used to control the growth of understory weeds and maintain the balance of the ecosystem (Tohiran et al., 2017).

Herbivores indirectly influence soil multifunctionality through changes in plant species diversity and plant functional structure. The effects of grazing on vegetation and soil can be rather complex in an unstable environment with low rainfall (Cheng et al., 2011). However, how grazing affects plant diversity and soil functioning in rainy and high temperature environment remains unclear. In our study, we utilized the density and traits of understory species, to measure soil carbon, nitrogen, and phosphorus content and enzyme activity associated with their transformation. Taxonomic diversity, functional diversity, and soil multifunctionality were calculated from the above measured data. We aimed to address the following questions: 1) how does grazing affect taxonomic diversity, functional diversity, and soil multifunctionality in tropical coconut plantations? and (2) how do plant-soil interactions reflect vegetation diversity (taxonomic diversity and functional diversity) and soil multifunctionality in a coconut plantation grazing system?

## 2 Materials and methods

### 2.1 Experimental design

**Figure 1:**
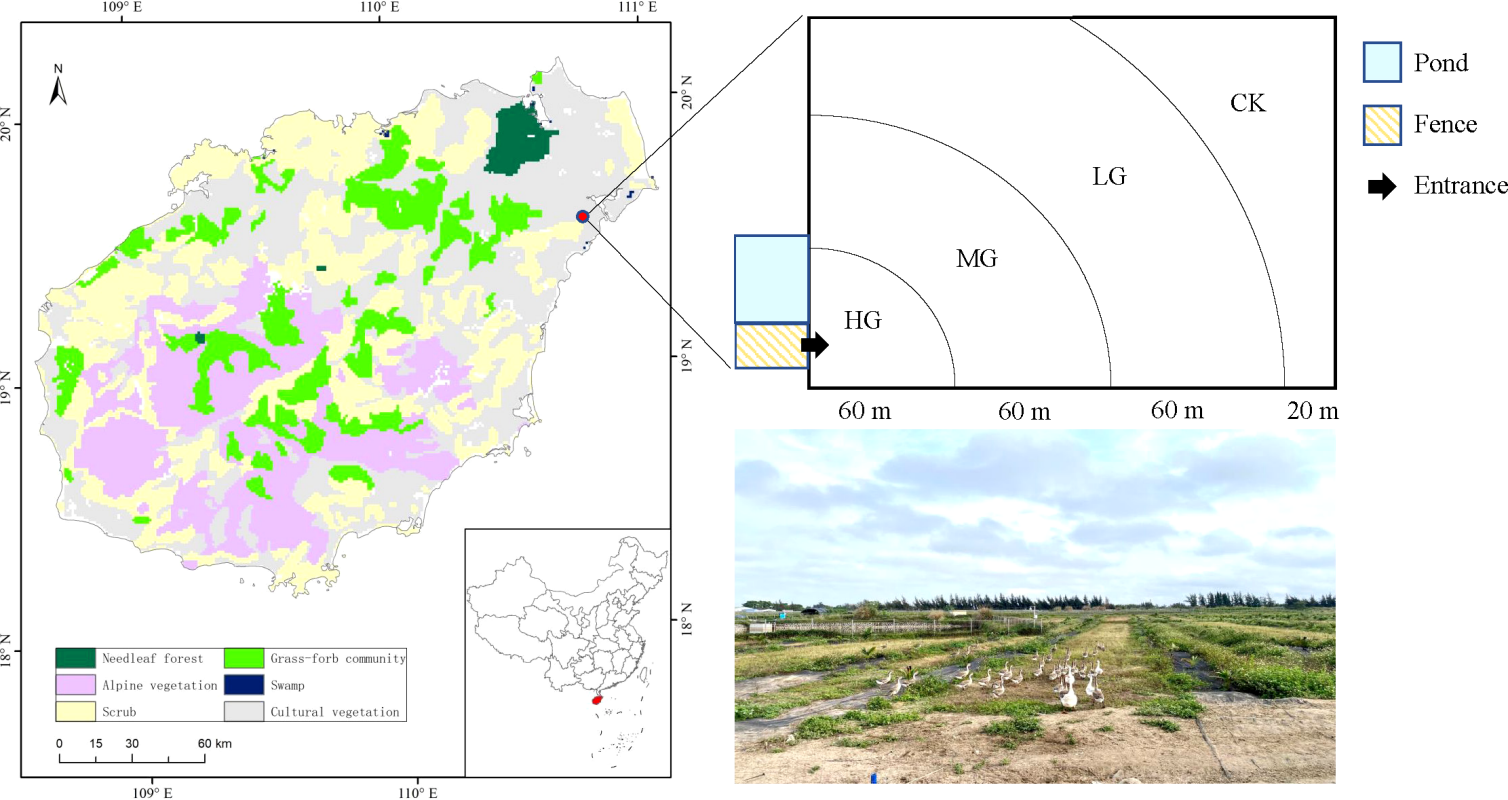
Study site and schematic representation of geese grazing intensities.

The study site was located in the four-team station of the Coconut Research Institute of Chinese Academy of Tropical Agricultural Sciences (19°32′37″N, 110°46′25″E, 144 m a.s.l.) in Wenchang City, Hainan Province, southern China (**Figure 1**). The average annual temperature in this region is 23.9°C, with a mean of 8,474.3 degree-d above 10°C. The total mean annual rainfall is 1,721.6 mm, 80% of which occurs from May to October, representing a tropical monsoon climate. The area has sandy soil (Gong et al., 2009). Tropical endemic coconuts are the main crop of the study area. The dominant species in the understory is *Bidens pilosa*, an invasive species. It is an annual herbaceous plant belonging to Asteraceae, with erect stems and a height of 30−100 cm, and propagates by seed, grows fast and can quickly cover other herbs.

Studies were conducted on a one-year-old coconut (*Cocos nucifera* cv. Wenye NO.4) flat plot plantation. The coconut plantation is an approximate rectangle of 200-m length and 150-m width. Coconut plants were arranged in double rows with 6-m row spacing, and plant spacing was 3−4 m. The grass grows freely in the coconut plantation, forming the understory grassland. The coconut plantation was fenced for goose grazing. From April 2021, the coconut plantation was grazed by 112 1-month-old geese. The pasture where geese graze should have sufficient forage resources and good grass quality. There must be a relatively flat pastoral road. The pasture should be as close to the pond as possible, where the geese can drink and bathe, and it should also be close to the goose house. The environment should be as quiet as possible to avoid frightening the geese. It is best to have shade trees or build a pergola in the pasture, so that the geese can be shaded in hot summer or sheltered from rain. Grazing in small pastures, the number of geese is controlled at 50-100. The age of the grazing geese should be the same. When the geese are one month old, they can graze all day long. At this stage, the geese grow rapidly and can make good use of green feed. Grazing can increase the amount of exercise of the geese, which is beneficial to the development of bones (Chen et al., 2018) In this study, geese were allowed to wander in coconut plantation by day and night and returned to the pond when they needed water. Therefore, grazing intensity was higher in places closer to the water source and lower in the places farther away. We identified four levels of grazing intensity at 0−60 m, 60−120 m, 120−180 m, and 180−200 m according to the aboveground biomass (Zhang et al., 2022). The corresponding grazing intensity was heavy grazing (HG), moderate grazing (MG), light grazing (LG), and no grazing (CK) (**Figure 1**). Six 1 m × 1 m quadrats were randomly selected for each grazing intensity in April 2022. In each quadrat, the density and number of fertile stems of each species were recorded. The height of five plants of each species in one quadrat was randomly measured (absolute plant height). In addition, the aboveground biomass of each species in each quadrat was clipped and transported back to the laboratory to dry and weigh (Hu et al., 2021). Soil samples were collected from four layers (0–10, 10–20, 20–30, and 30–40 cm) after the aboveground green plants and litter were collected. We did this by randomly taking two cores from the same layer of soil in each square and pooling them as a composite sample.

### 2.2 Experimental measurements

The Walkley-Black dichromate oxidation method was used to determine soil organic matter (SOM) (Walkley and Black, 1934). The Kjeldahl method was used to determine soil total nitrogen (TN) (Pruden et al., 1985). Mo-Sb colorimetric method was used to determine total phosphorus (TP) (Murphy and Riley, 1962). A flow injection auto-analyzer was used to determine ammonium nitrogen ( ${\text{NH}}_{4}^{+}$ -N) and nitrate nitrogen ( ${\text{NO}}_{3}^{-}$ -N) content (Sah, 1994). Spectrophotometry was performed to analyze the rapidly available phosphorus (AP) after extraction with 0.5 mol·L^−1^ NaHCO_3_ (Olsen, 1954).

Urease, acidic phosphatase, and β-glucosidase activities were analyzed colorimetrically according to Caravaca et al. (2005).

## 3 Data analysis

### 3.1 Vegetation diversity index and soil function index

Taxonomic diversity was calculated using the density of each species, and functional diversity was estimated using the nine traits in each quadrat (**Table S1**). Before using plant traits to calculate functional diversity and soil properties to calculate soil functions, *z*-transformation was employed to standardize data.

Taxonomic diversity was represented by species richness (*S*), Shannon’s diversity (*H*), and Pielou index (*E*). Functional diversity was represented by modified functional attribute diversity (MFAD), functional divergence (Fdiv), and functional evenness (Feve), and these indices are described in detail in our previous study (Hu et al., 2021). A simple description is as follows:


$$H=\;-\sum_{i}^{S}p_{i}\ln(p_{i}),$$

E=H/lnS,

where *p_i_* is the ratio of the density of *i* species to the density of all species in the quadrat, and *S* is the total number of species in the community.


$$MFAD=(\sum_{h=1}^{N}\sum_{k=1}^{N}d_{hk})/N;$$

$$d_{hk}=\sum_{i=1}^{p}\left|a_{hi}a_{ki}\right|/\sum_{i=1}^{p}max\left\{a_{hi},a_{ki}\right\},$$

$$Fdiv\;=\sum_{h=1}^{N}\sum_{k>1}^{N}d_{hk}p_{h}p_{k},$$

$$Feve=\left\{\sum_{b=1}^{S-1}\min\left(PEW_{b},1/(S-1\right))-1/\left(S-1\right)\}/\{1-1/\left(S-1\right)\right\},$$

$$PEW_{b}=EW_{b}/\sum_{b=1}^{S-1}EW_{b},$$

$$EW_{b}=d_{hk}/(p_{h}+p_{k}),$$

where *N* is the number of functional traits, *d_hk_* is the dissimilarity between functional traits *h* and *k*, *a_hi_* is the affinity of functional trait *h* to trait *i*, and *a_ki_* is the affinity of functional trait *k* to trait *i*.

Soil functions were calculated by C functioning index (OM and β‐glucosidase), N functioning index (TN, ${\text{NO}}_{3}^{-}$ -N, ${\text{NH}}_{4}^{+}$ -N, and urease), P functioning index (TP, AP and acidic phosphatase), and overall soil multifunctionality index (the above nine soil properties) (Maestre et al., 2012; Delgado-Baquerizo, 2016).

### 3.2 Statistical analysis

**Figure 2:**
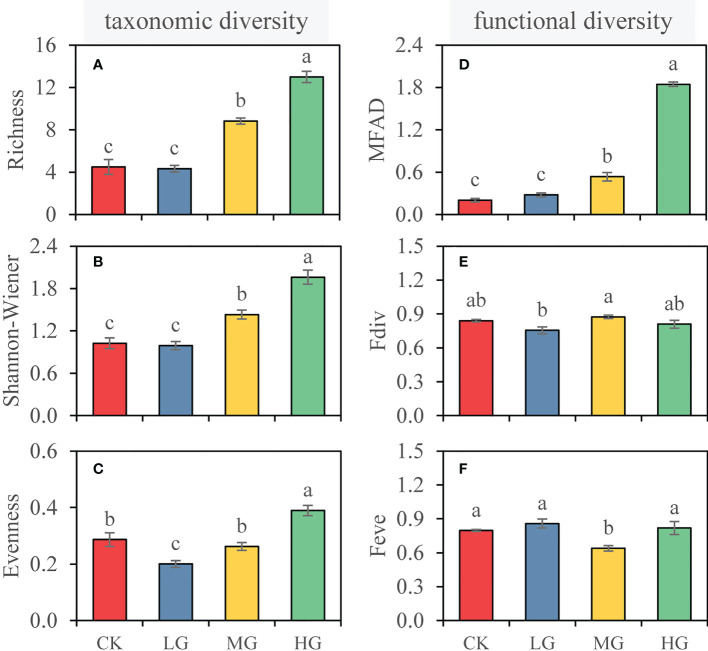
Taxonomic diversity (left) and functional diversity (right) of each grazing intensity. **(A)** Species richness, **(B)** Shannon-Wiener index, **(C)** species evenness, **(D)** modified functional attribute diversity (MFAD), **(E)** functional divergence (Fdiv), and **(F)** functional evenness (Feve). Values represent the mean ± SE. Different letters represent significant differences among grazing intensities at the *p* = 0.05 level. (CK, no grazing; LG, light grazing; MG, moderate grazing; HG, heavy grazing).

**Figure 3:**
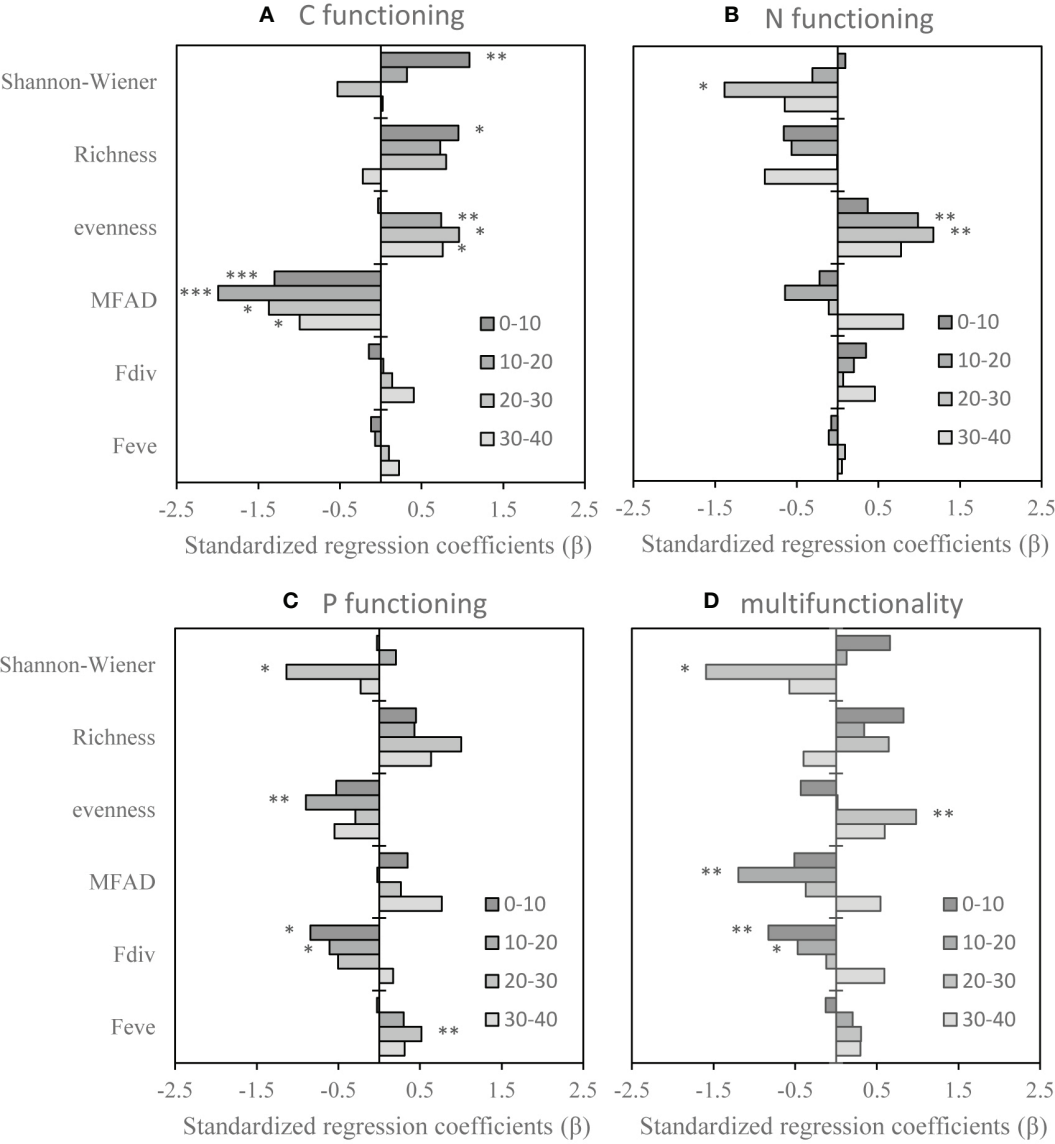
Standardized regression coefficients (β) obtained from multiple linear mixed effect models (LMMs) for each soil functioning in four depths. **(A)** Carbon functioning, **(B)** nitrogen functioning, **(C)** phosphorus functioning, **(D)** multifunctionality. MFAD, modified functional attribute diversity; Fdiv, functional divergence; Feve, functional evenness. ***, **, and * stand for *P* < 0.001, *P* < 0.01, and *P* < 0.05, respectively.

One-way analysis of variance was used to determine differences in taxonomic diversity (species richness, Shannon-Wiener index, species evenness) and functional diversity (MFAD, functional divergence, functional evenness) among four grazing intensities. Statistical significance was considered at *P* < 0.05. Mean values (± SE) are presented in **Figure 2**. As data were not normally distributed (Shapiro-Wilk test), a generalized linear model (GLIMMIX procedure) was applied to quantify the effects of grazing intensity (G) and soil depth (S) on the soil properties (**Table 1**). The model is y = G + S + G×S + Γ+ ϵ, where Γ is the random effect of replicate, and ϵ is the model error. All data were analyzed using SAS software (SAS 9.4, SAS Institute Inc., Cary, NC, USA). The rejection level of H0 was set at *P* < 0.05. Furthermore, to examine the effects of plant diversity (taxonomic diversity and functional diversity) on the soil functions (C functioning, N functioning, P functioning, soil multifunctionality), we performed a series of multiple linear regressions models. We obtained the standardized regression coefficient (β) of each predictor (**Figure 3**).

**Table 1:** Effects of grazing intensity and soil layer on the soil properties, mean value ± SE, n = 6 subplots, with statistical results of the general linear model (*P* value).

Grazing intensity	Soil depth (cm)	OM (%)	TN (g kg^−1^)	${\text{NH}}_{4}^{+}$ -N (mg kg^−1^)	${\text{NO}}_{3}^{-}$ -N (mg kg^−1^)	TP (g kg^−1^)	AP (mg kg^−1^)	Urease mg/(24 h ×g)	β‐glucosidase μmol/(24 hxg)	Acidic phosphatase μmol/(24 h×g)
CK	0-10	0.91 ± 0.02	0.41 ± 0.01	3.9 ± 0.6	1.9 ± 0.04	0.15 ± 0.01	7.9 ± 0.4	0.65 ± 0.01	4.0 ± 0.8	5.9 ± 0.2
	10-20	1.11 ± 0.02	0.44 ± 0.01	7.7 ± 0.3	1.2 ± 0.05	0.11 ± 0.01	10.8 ± 0.5	0.40 ± 0.01	2.0 ± 0.3	3.6 ± 0.1
	20-30	1.37 ± 0.03	0.55 ± 0.00	6.9 ± 1.6	4.7 ± 0.21	0.22 ± 0.01	4.3 ± 0.1	0.27 ± 0.02	1.3 ± 0.1	2.4 ± 0.1
	30-40	1.34 ± 0.02	0.48 ± 0.00	7.4 ± 1.4	3.2 ± 0.29	0.17 ± 0.01	0.0 ± 0.0	0.21 ± 0.04	1.0 ± 0.1	1.9 ± 0.2
LG	0-10	0.79 ± 0.01	0.32 ± 0.01	5.8 ± 1.0	1.9 ± 0.04	0.23 ± 0.01	31.3 ± 4.1	0.60 ± 0.01	4.1 ± 0.7	7.9 ± 0.3
	10-20	0.83 ± 0.04	0.29 ± 0.02	2.3 ± 0.2	1.8 ± 0.12	0.23 ± 0.01	31.3 ± 4.1	0.36 ± 0.00	2.0 ± 0.3	4.7 ± 0.2
	20-30	0.88 ± 0.07	0.42 ± 0.02	5.6 ± 1.2	2.5 ± 0.27	0.24 ± 0.03	15.3 ± 2.9	0.24 ± 0.02	1.4 ± 0.3	3.2 ± 0.2
	30-40	0.79 ± 0.09	0.34 ± 0.03	1.8 ± 0.4	2.7 ± 0.11	0.16 ± 0.01	7.6 ± 1.1	0.18 ± 0.04	1.0 ± 0.3	2.4 ± 0.3
MG	0-10	0.94 ± 0.02	0.38 ± 0.01	2.2 ± 0.4	2.0 ± 0.15	0.17 ± 0.01	18.1 ± 1.2	0.62 ± 0.01	13.4 ± 0.8	6.8 ± 0.3
	10-20	0.84 ± 0.02	0.30 ± 0.01	6.2 ± 0.7	0.8 ± 0.07	0.15 ± 0.00	11.1 ± 3.1	0.38 ± 0.00	6.7 ± 0.4	4.1 ± 0.2
	20-30	1.06 ± 0.10	0.39 ± 0.06	7.1 ± 1.1	1.3 ± 0.05	0.14 ± 0.01	10.0 ± 2.2	0.25 ± 0.02	4.5 ± 0.3	2.7 ± 0.1
	30-40	0.86 ± 0.07	0.27 ± 0.04	6.1 ± 1.7	2.1 ± 0.09	0.16 ± 0.00	11.8 ± 2.0	0.20 ± 0.03	3.5 ± 0.3	2.1 ± 0.2
HG	0-10	0.71 ± 0.01	0.30 ± 0.02	4.8 ± 0.5	1.8 ± 0.03	0.28 ± 0.03	16.4 ± 3.3	0.59 ± 0.02	14.4 ± 1.7	7.6 ± 0.1
	10-20	0.49 ± 0.01	0.15 ± 0.03	3.6 ± 0.8	1.7 ± 0.21	0.19 ± 0.01	6.0 ± 0.9	0.35 ± 0.01	6.7 ± 0.4	4.6 ± 0.1
	20-30	0.72 ± 0.05	0.29 ± 0.04	10.2 ± 1.7	1.6 ± 0.11	0.21 ± 0.03	8.5 ± 0.7	0.23 ± 0.02	4.5 ± 0.3	3.1 ± 0.2
	30-40	0.52 ± 0.02	0.16 ± 0.04	14.1 ± 1.8	2.7 ± 0.13	0.22 ± 0.03	19.5 ± 1.3	0.18 ± 0.03	3.4 ± 0.2	2.4 ± 0.3
Grazing intensity (G)		0.000	0.000	0.000	0.000	0.000	0.000	0.060	0.000	0.000
Soil depth (S)		0.000	0.000	0.000	0.000	0.013	0.000	0.000	0.000	0.000
G×S		0.000	0.022	0.000	0.000	0.003	0.000	1.000	0.000	0.164

We also used non-metric multidimensional scaling (NMDS) to describe the composition of the plant community in four grazing intensities. NMDS was conducted in R version 4.3 (R Development Team, 2013).

## 4 Results

### 4.1 Community composition under coconut palms

**Figure 4:**
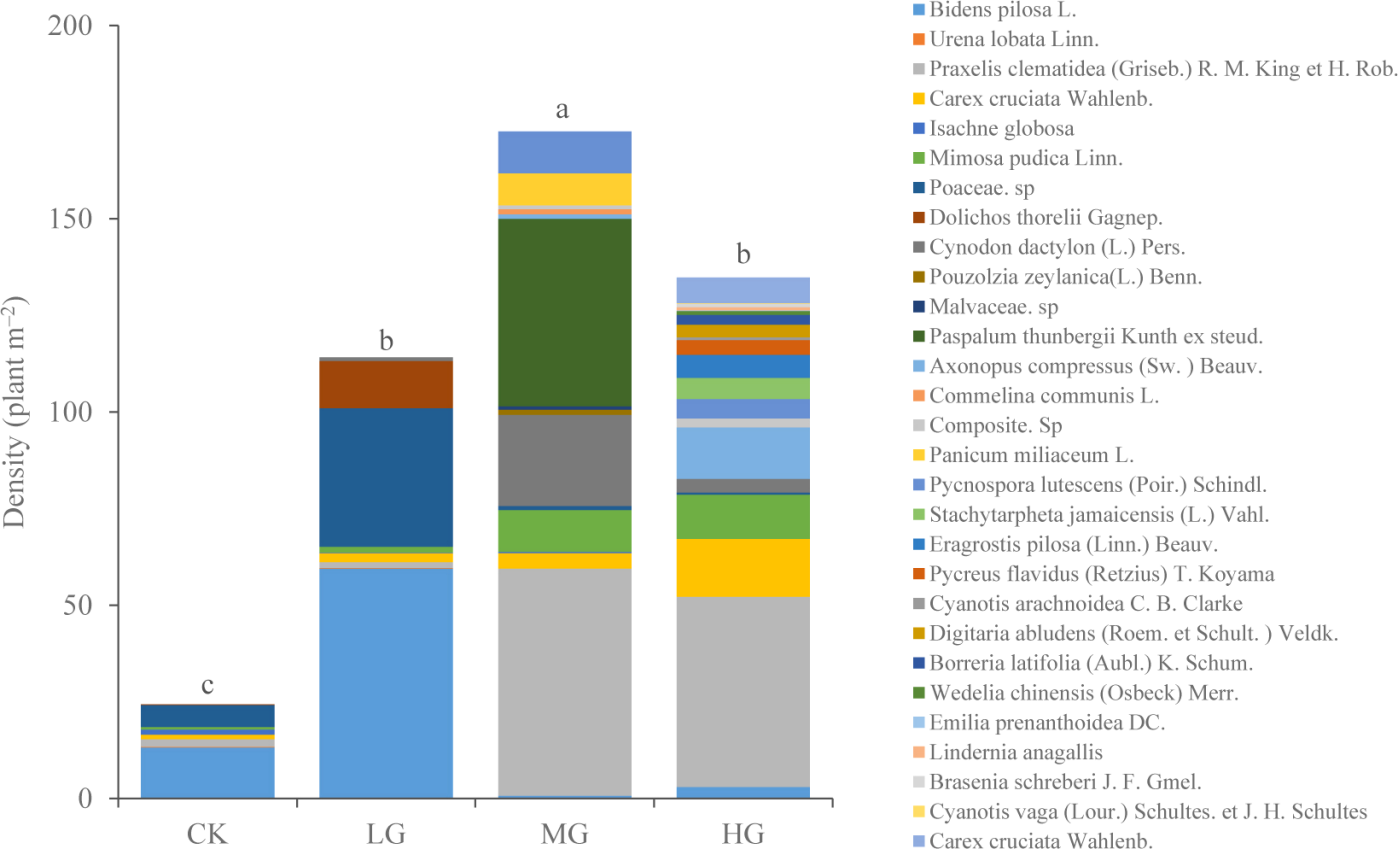
Aboveground species composition under different grazing intensities. The mean density of each species (n = 6). Different letters represent significant differences in total density among grazing intensities at the *P* = 0.05 level. (CK, no grazing, LG, light grazing, MG, moderate grazing, HG, heavy grazing).

**Figure 5:**
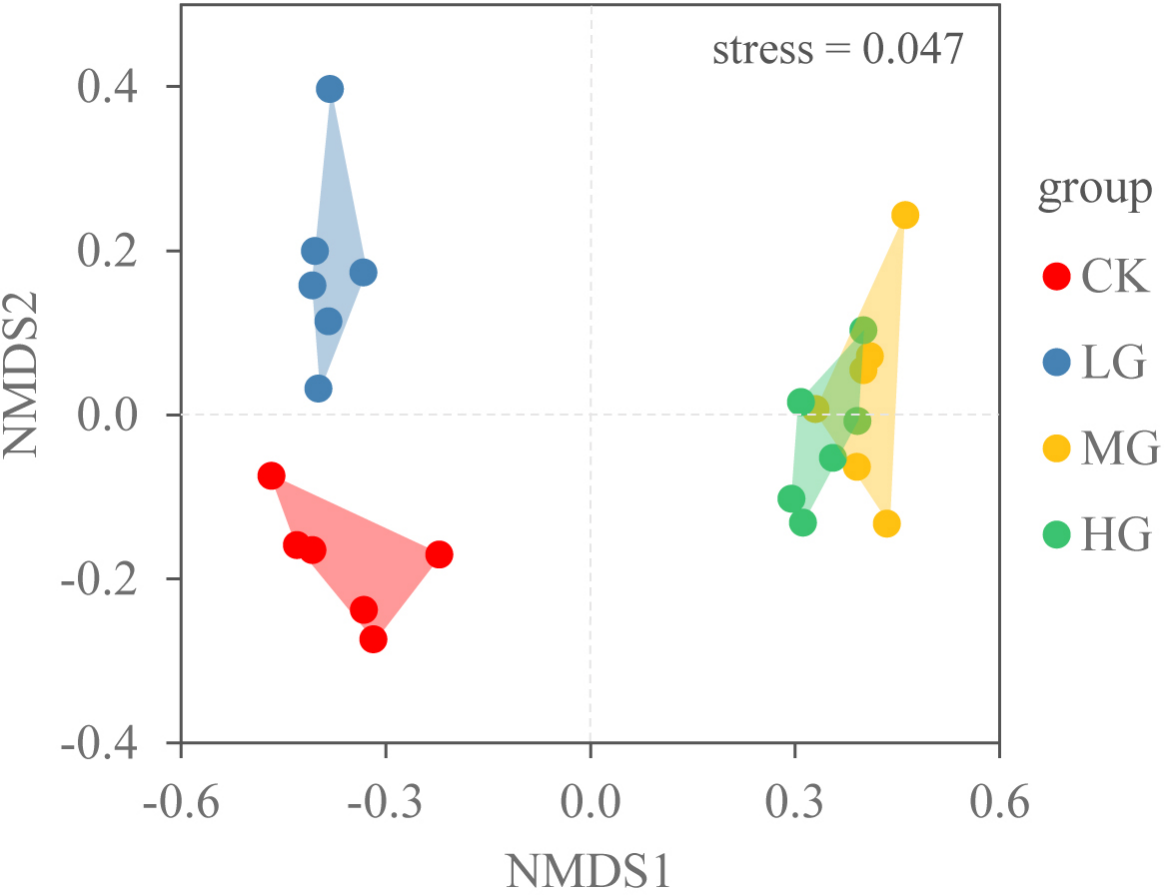
Ordination diagram for four grazing intensities (CK: no grazing, LG: light grazing, MG: moderate grazing, HG: heavy grazing) for the aboveground species density using non-metric multidimensional scaling NMDS.

Grazing has reshaped understory community structure (**Figures 4**, **5**). In CK and LG treatments, *B. pilosa* was the predominant species in the understory community, while in MG treatment, *Praxelis clematidea* + *Paspalum thunbergii* was the predominant species. However, in HG, only the *P. clematidea* was the predominant species. Grazing effectively inhibited the growth of *B. pilosa* (invasive weed) but increased the growth of *P. clematidea* (invasive weed) and *P. thunbergii* (high-quality forage) in MG.

The NMDS plot of total density revealed that the community composition under each grazing intensity was clustered, with a small overlap between MG and HG treatments (**Figure 5**). With the increasing grazing intensity, plant density initially increased and subsequently decreased. They were only 24 plants·m^−2^ in CK. The maximum vegetation density was 173 plants·m^−2^ in MG (*P* < 0.05) (**Figure 4**).

### 4.2 Taxonomic diversity and functional diversity

The species richness of CK was 4.5, but it did not significantly respond to light intensity grazing (*P* > 0.05; **Figure 2A**). However, species richness significantly increased in MG (8.8) and HG (13.0). Shannon-Wiener index showed the same response to grazing intensity as richness and was 1.02, 0.99,1.43, and 1.96 in CK, LG, MG, and HG, respectively (**Figure 2B**). Compared with CK, LG significantly reduced species evenness by 30%, while MG maintained species evenness (0.26), and HG significantly increased species evenness (0.39) (**Figure 2C**).

The MFAD of CK was 0.20. The change in MFAD was not affected by LG. With increasing grazing intensity, both MG and HG significantly increased MFAD, and the highest in HG was 1.84 (**Figure 2D**). However, we found that grazing in coconut plantation had no significant effect on Fdiv, regardless of grazing intensity (**Figure 2E**). Similarly, grazing had minimal effects on Feve, and MG significantly reduced Feve (*P* < 0.05; **Figure 2F**).

### 4.3 Soil properties and soil functions

Grazing intensity and soil depth, as well as their interactions, had significant effects on soil OM, TN, ${\text{NH}}_{4}^{+}$ -N, ${\text{NO}}_{3}^{-}$ -N, TP, and AP content (**Table 1**). In CK, we found that OM content increased with soil depth, while grazing weakened the effect of soil depth on soil OM content. Moreover, we found that soil OM and TN content in each soil layer decreased with increasing of grazing intensity. Soil ${\text{NH}}_{4}^{+}$ -N and ${\text{NO}}_{3}^{-}$ -N content increased with soil depth, but the increase in ${\text{NH}}_{4}^{+}$ -N and ${\text{NO}}_{3}^{-}$ -N content showed large and small changes, respectively, when grazing intensity was higher. Grazing promoted the increase in AP content in all soil layers. In addition, soil AP decreased with increasing soil depth, except in heavily grazed land (**Table 1**).

Soil urease activity was significantly affected by soil layer (*P* < 0.001); grazing and the interaction between grazing and soil layer had no significant effect on urease activity (both *P* > 0.05). Grazing, soil layer, and their interaction had significant effects on β‐glucosidase activity. Grazing and soil layer had significant effects on acidic phosphatase, but their interaction was not statistically significant (*P* > 0.05). The activities of urease, β‐glucosidase, and acidic phosphatase decreased with increasing soil depth. The activity of soil urease decreased with increasing grazing intensity. MG and HG significantly increased soil β‐glucosidase activity. Grazing increased the activity of acidic phosphatase in the 0–10-cm and 10–20-cm soil layers. However, the effect was small for the 20–30-cm and 30–40-cm soil layers (**Table 1**).

**Figure 6:**
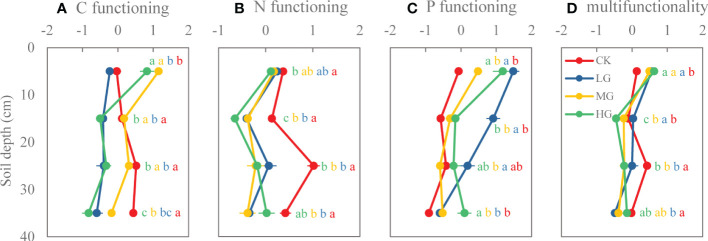
Soil functions of different soil layers under various grazing intensities. **(A)** Carbon functioning, **(B)** nitrogen functioning, **(C)** phosphorus functioning, **(D)** multifunctionality. Different lowercase letters represent significant differences among grazing intensities at the *P* = 0.05 level. (CK: no grazing, LG: light grazing, MG: moderate grazing, HG: heavy grazing).

LG decreased C functioning of each soil layer. MG and HG increased C functioning of the 0−10-cm soil layer, but decreased C functioning in 10−20-cm, 20−30-cm, and 30−40-cm layers. In addition, C functioning of soil layers in MG was higher than that of soil layers in LG and HG (**Figure 6A**). Compared with CK, grazing significantly reduced soil N functioning in each soil layer, and with increasing soil depth, stronger negative effects of grazing on N functioning were observed (**Figure 6B**). Grazing increased P functioning of soil in each layer, but the effect decreased with soil depth (**Figure 6C**). Grazing significantly increased soil multifunctionality at the 0−10-cm soil layer, and there was no significant difference among grazing intensities. However, grazing reduced soil multifunctionality with increasing soil depth (**Figure 6D**).

### 4.4 Relationship between vegetation diversity and soil function

The response of soil function to vegetation diversity was influenced by soil depth. Soil C functioning in the 0−10-cm soil layer was positively affected by the Shannon-Wiener index and richness (standardized regression coefficients: β = 1.1 and β = 1.0, respectively), while it was negatively affected by MFAD (−1.3). Soil C functioning in 10−20-cm soil layer was affected by evenness and MFAD (β = 0.7 and β = −2.0, respectively. Soil C functioning in 20−30-cm and 30−40-cm soil layers was affected by evenness (β = 1.0 and β = 0.8, respectively) and MFAD (β = −1.3 and β = −1.0, respectively) (**Figure 3A**). Soil N functioning in the 0−10-cm soil layer was not affected by vegetation diversity. N functioning in the 10−20-cm soil layer was affected by evenness (β = 1.0). N functioning of the 20−30-cm soil layer was affected by both the Shannon-Wiener index and evenness (β = −1.4 and β = 1.2, respectively) (**Figure 3B**). Soil P functioning in the 0−10-cm soil layer was affected by Fdiv (β = −0.8). P functioning in the 10−20-cm soil layer was affected by both evenness and Fdiv (β = −0.9 and β = −0.6, respectively), while P functioning in the 20−30-cm soil layer was affected by Feve (β = −0.5) (**Figure 3C**). Soil multifunctionality in the 0−10-cm soil layer was negatively affected by Fdiv (β = −0.8). Soil multifunctionality in the 10−20-cm soil layer was negatively affected by MFAD and Fdiv (β = −1.2 and β = −0.5, respectively). Soil multifunctionality in the 20−30-cm soil layer was affected by the Shannon-Wiener index and evenness (β = −1.6 and β = 1, respectively) (**Figure 3D**).

## 5 Discussion

### 5.1 Effects of grazing on vegetation diversity

Our main objective was to examine the effect of grazing on the vegetation diversity, including taxonomic diversity and functional diversity, and soil functions in a tropical coconut plantation. Our study was conducted in a coconut plantation that was grazed by geese for 1 year. However, some previous studies have pointed out that the impact of grazing on species diversity was relatively small compared with the impact of environmental variation (Christensen et al., 2004). Ren et al. (2012) reported that grazing intensity had a weak effect on vegetation composition and diversity after short-term grazing. However, in this study, we found that grazing altered community structure (**Figures 4**, **5**), particularly after moderate and heavy grazing, and significantly changed the taxonomic diversity (**Figures 2A−C**). For functional diversity, we only found that MFAD had a significant response to grazing intensity (**Figure 2D**), but neither Fdiv nor Feve had a significant response to grazing (**Figure 2E, F**). This study utilized a year-round grazing system with geese grazing at all times of the day and night, which has been described in detail Materials and Methods. However, many studies on grazing focused on temperate grassland areas (Hu et al., 2019) or even further north, where grazing occurred for only half a year or even less (2−3 months) each year and in the day time, with the return to the corral at night. In Central Apennines, grazing sheep for 4 months (June to the end of September) per year in a 30-year grazing system significantly reduced grassland species diversity and functional diversity (Catorci et al., 2014). In Madrid, Spain, Carmona et al. (2012) reported that grazing intensity had significant effects on taxonomic diversity and functional diversity of plots grazed for about 30 years.

In China, research on grazing is mainly concentrated in the northern region, and the grassland area is very small in the south; so, there are few grazing studies, especially in the tropical regions. However, there is a large understory area in tropical areas, especially in Hainan, which is the main coconut-producing area in China. There are sufficient understory resources, and the free understory area is 5−8 times the area of coconut plantation. Due to better hydrothermal conditions, understory vegetation grows faster with higher yields and could carry more livestock than traditional grazing areas in northern China. In addition, the geese in this study were free to feed both day and night and were not driven back to the pen; so, the grazing pressure on grassland was 5−10 times that on the traditional grazing areas in northern China, which may change the grassland community and the taxonomic diversity after 1 year of grazing.

Alterations in vegetation functional diversity not only change the structure of aboveground species community but also influence the traits of various species, such as flowering time, flowering period, or species morphological characteristics, plant height, and biomass (Peco et al., 2017). This may take longer to change, and much depends on factors such as the environment (Carmona et al., 2012). In this study, plant functional diversity MFAD was affected by grazing intensity, while Fdiv and Feve showed little response to grazing. This indicates that the grazing intensity in this study has a significant effect on the dispersion of species in the functional traits space. The Fdiv ranged from 0.75 to 0.87 in this study, with small fluctuations (**Figure 2E**), indicating that grazing intensity had minimal effects on the rate of changes in functional traits, and did not significantly affect specific traits of some species. Similarly, Feve values in this study ranged from 0.64 to 0.86, and the minimum was in moderate grazing, while there was no significant difference between low and high grazing intensity and CK (**Figure 2F**), indicating that moderate grazing reduces the functional traits of some species. This is concordant to the findings of Ford et al. (2018) in northwest Wales, UK, where an intensive grazing (managed sheep or feral goat) system was built for at least the past 25 years, and functional diversity did not differ with grazing intensity for understory plants. There are also some studies showing that high grazing intensity can reduce functional diversity (Carmona et al., 2012). However, in general, it takes time for grazing to shape the functional diversity of vegetation, which may be related to the environment and the direction of community succession (Carmona et al., 2012; Hu et al., 2021).

### 5.2 Effects of grazing on soil function

Grazing utilization leads to heterogeneity in aboveground vegetation composition and soil physicochemical conditions, and provides different soil functions through processes such as interactions between aboveground and belowground species (Eldridge et al., 2019). With the deepening of the understanding of soil functions, researchers have gradually realized that soil can have multiple functions and services at the same time such as C functioning, N functioning, P functioning and multifunctionality (Zwetsloot et al, 2021). Grazing intensity affects soil nutrients and physicochemical properties by increasing trampling, defoliation, and manure return, resulting in unpredictable changes in soil function (Cislaghi et al, 2019). In this study, grazing significantly increased C functioning, P functioning, and multifunctionality in the 0−10-cm soil layer, but decreased N functioning in the 0−10-cm soil layer, but with increasing soil depth, grazing had a negative cumulative effect on soil multifunctionality (**Figure 6**). This may be due to the fact that grazing increased the material transformation capacity of the topsoil and the enzyme activity of the topsoil, and at the same time, it transports nutrients from the bottom soil to the topsoil (0−10-cm soil layer), increasing C functioning, P functioning, and multifunctionality of the topsoil. In perennial grazing plots, urine and manure input increases soil active organic carbon and organic nitrogen input (Harrison and Bardgett, 2008). Grazing increases root-to-shoot ratio and root exudates and possibly increases primary grassland productivity, thereby increasing the utilization of belowground active organic carbon and C functioning (Peco et al., 2017). However, because goose excrement mainly exists in the form of N compounds and covers the surface soil, the function of N in the soil surface (0−10-cm soil layer) is inhibited. The precipitation in tropical regions is rich, and excess N compounds are leached into the deep soil, which may further reduce the originally lower N functioning.

### 5.3 Effects of vegetation diversity on soil function

This study is a novel attempt to assess the taxonomic diversity and functional diversity that mediate soil functions in response to grazing in the understory of a coconut plantation. As expected, we found that taxonomic diversity and functional diversity in this study more or less mediate effects on soil functions (C functioning, N functioning, P functioning, and multifunctionality). However, these effects vary with soil layer and were regulated by grazing (**Figure 3**). It has been previously reported that the effect of grazing on nutrient cycling, i.e., soil function, depends on primary productivity (Peco et al., 2017).

In this study, we found that the significant effects of taxonomic diversity on soil C functioning were all positive, and only MFAD had a significant negative effect on C functioning in all soil layers (**Figure 3A**). This was influenced by grazing, which had a significant effect on the richness, Shannon-Wiener index, and evenness of taxonomic diversity and only had a significant effect on MFAD. In addition, we believe that the transformation of C is still greatly affected by the aboveground community, which is concordant to the results of Harrison and Bardgett (2008). The aboveground vegetation is affected by grazing that in turn changes the community structure, alters their traits, and also modifies their utilization and absorption of soil nutrients. This also confirms the relationship between aboveground vegetation diversity and soil C functioning. We found that the transformation of N was affected by taxonomic diversity, but not by functional diversity, indicating that regardless of how the aboveground vegetation communities changed and how their traits changed, their transformation and utilization of N are relatively stable, and only a few changes will occur. However, it is undeniable that changes in taxonomic diversity and functional diversity have not reached a threshold that can alter N functioning. It is possible to increase the grazing years to significantly change the taxonomic and functional diversities. In addition, we found that both taxonomic diversity and functional diversity have a significant impact on the function of P, and some studies have shown that soil P content in tropical regions is very low and thus is P-limited (Hu et al., 2022). Therefore, a slight change in the aboveground vegetation will change P functioning. Although we found that grazing had no significant effect on Fdiv and Feve, small changes in Fdiv had a significant negative effect on P functioning at 0−10-cm and 10−20-cm soil layers; whereas changes in Feve had a positive effect on P functioning. Soil multifunctionality is the comprehensive performance of various soil functions. The response of soil multifunctionality to functional traits MFAD and Fdiv was negative, indicating that the diversity of functional traits of aboveground vegetation increases, and the large difference in traits would reduce soil multifunctionality. It is not conducive to soil health, while the increase in the evenness is beneficial to community stability and increases soil multifunctionality.

In general, changes in vegetation taxonomic diversity and functional diversity are regulated by a various factor, and their impact on soil function is also regulated by the comprehensive regulation of plant species and traits. Grazing is the main factor that changes the interactions between vegetation and soil. Moderate grazing, in particular, is the key to a balanced forest-grass-herbivore ecosystem. Therefore, it is necessary to conduct long-term research on quantitative grazing, to explore the relationship between vegetation diversity and soil functions in coconut plantation in tropical regions.

## 6 Conclusion

The aim of this study was to assess the community structure and diversity, as well as changes in soil functions, and their relationships through geese grazing in a coconut plantation in the tropics. We found that grazing largely impacted community structure. With the increasing grazing intensity, the community with the predominant species being *B. pilosa* changed to the community with the predominant species being *P. clematidea* + *P. thunbergii* and finally changed to the community with the predominant species being *P.* clematidea. The taxonomic diversity strongly responded to moderate and heavy grazing intensities, showing an increasing trend. Among the functional diversity, only MFAD responded strongly to moderate and heavy grazing intensity, while Fdiv and Feve had no significant response to grazing intensity. Grazing increased C functioning, P functioning, and multifunctionality but reduced N functioning in the surface 0−10-cm soil layer. However, with increasing soil depth, grazing reduced the various soil functions. Through multiple regression analysis, we found that vegetation taxonomic diversity and functional diversity can be used to fit soil functions in different soil layers. The increase of taxonomic diversity was beneficial to increase soil C functioning in each soil layer, while MFAD had a significant negative effect on soil C functioning in each soil layer. Functional diversity had no significant effect on soil N functioning, while the Shannon-Wiener index and richness both had negative effects on N functioning, and increased evenness could promote N functioning. The Shannon-Wiener index, evenness, and Fdiv are negative for P functioning, while Feve is positive for P functioning. The Shannon-Wiener index, MFAD, and Fdiv all had negative effects on soil multifunctionality, while evenness could promote the increase of soil multifunctionality in deeper soil layers. The relationship between aboveground vegetation diversity and soil functions in tropical regions is complex and is affected by grazing intensity and different soil depths. To quantify their relationship in future studies, it is necessary to quantify grazing intensity and increase grazing years for further research.

## Data availability statement

The datasets presented in this study can be found in online repositories. The names of the repository/repositories and accession number(s) can be found below: https://figshare.com/, https://doi.org/10.6084/m9.figshare.21590607.v1.

## Author contributions

AH, XL, RD, GL designed the program of studies and experiments, guided the entire process of the study, and revised the manuscript. AH, XL, RD, WY, RY finished the material collection. QD, AH, HH, carried out most of the nutrient analysis. QD and AH analysed the data, and wrote the paper. All authors read and approved the content of the paper. All authors contributed to the article and approved the submitted version.
