# Supplementary material for: Effects of grazing on vegetation diversity and soil multifunctionality in coconut plantations
Source: Front Plant Sci. 2023 Jan 11;13:1109877. doi: 10.3389/fpls.2022.1109877 (PMC9874161; doi:10.3389/fpls.2022.1109877)
Supplement: Supplementary file 1 [file Table_1.docx]

Table S1 Tan plant traits were used to calculate the functional diversity

| Traits | Attach values to each trait |
| --- | --- |
| Aboveground biomass | Each species biomass per square meter |
| Species density | Each species density per square meter |
| Reproductive branch number | The average reproductive branch number of each species per square meter |
| Plant height | The average plant height of each species per square meter |
| Life form | Annual: 1; Perennial: 2 |
| Classification | Herbaceous: 1; liana: 2; Semi-shurb: 3 |
| Nitrogen fixation | Yes: 1; No: 2 |
| Bloom time | Which month go to blooms |
| Bloom periods | Months of flowering |
